# Blood Feeding Sources of *Nyssomyia antunesi* (Diptera: Psychodidae): A Suspected Vector of *Leishmania* (Kinetoplastida: Trypanosomatidae) in the Brazilian Amazon

**DOI:** 10.1093/jme/tjac108

**Published:** 2022-07-28

**Authors:** Amanda Costa Pimentel, Yetsenia del Valle Sánchez Uzcátegui, Ana Carolina Stocco de Lima, Fernando Tobias Silveira, Thiago Vasconcelos dos Santos, Edna Aoba Yassui Ishikawa

**Affiliations:** Programa de Pós Graduação em Doenças Tropicais, Núcleo de Medicina Tropical, Universidade Federal do Pará, Belém, Pará State, Brazil; Programa de Pós Graduação em Biologia de Agentes Infecciosos e Parasitários, Instituto de Ciências Biológicas, Universidade Federal do Pará, Belém, Pará State, Brazil; Seção de Parasitologia, Instituto Evandro Chagas, Ananindeua, Pará State, Brazil; Departamento de Biología, Facultad de Ciencias, Universidad de Los Andes, Mérida, Venezuela; Seção de Parasitologia, Instituto Evandro Chagas, Ananindeua, Pará State, Brazil; Programa de Pós Graduação em Doenças Tropicais, Núcleo de Medicina Tropical, Universidade Federal do Pará, Belém, Pará State, Brazil; Seção de Parasitologia, Instituto Evandro Chagas, Ananindeua, Pará State, Brazil; Programa de Pós Graduação em Biologia de Agentes Infecciosos e Parasitários, Instituto de Ciências Biológicas, Universidade Federal do Pará, Belém, Pará State, Brazil; Seção de Parasitologia, Instituto Evandro Chagas, Ananindeua, Pará State, Brazil; Programa de Pós Graduação em Doenças Tropicais, Núcleo de Medicina Tropical, Universidade Federal do Pará, Belém, Pará State, Brazil

**Keywords:** Phlebotominae, host, mammal, hematophagous insect, transmission

## Abstract

Present work aimed to identify blood feeding sources and attempt to detect *Leishmania* DNA in *Nyssomyia antunesi*, suspected vector of *Leishmania* sp., from a park in the urban center of Belém, the capital of Pará State, in the Brazilian Amazon. Entire bodies and gut contents of *Ny. antunesi* engorged females, previously captured in the urban park with Centers for Disease Control (CDC) light traps and aspiration on tree bases, were subjected to *Leishmania* and vertebrate DNA detection through amplification of the *Leishmania* mini-exon and vertebrate cytochrome b (cyt b) gene regions, respectively. The quality of DNA extraction from entire bodies was ensured through amplification of the dipteran cyt b region. The vertebrate cyt b amplicons were sequenced and compared with those available on GenBank. A maximum likelihood phylogenetic tree was constructed to assess the clustering patterns of these sequences. *Leishmania* DNA was not detected. The sequences of 13 vertebrate cyt b amplicons were considered informative, exhibiting similarity and clustering with the following six vertebrate species: *Dasyprocta leporina* (1), *Cuniculus paca* (1), *Tamandua tetradactyla* (4), *Choloepus didactylus* (4), *Pteroglossus aracari aracari* (2), *Homo sapiens* (1). The samples of *D. leporina* and *C. paca* were obtained from the CDC canopy, whereas the others were by aspiration from tree bases. The present results revealed the eclectic and opportunist blood-feeding behavior of *Ny. antunesi*, with birds and mammals, these last ones acting as potential reservoirs for *Leishmania* species, distributed throughout the vertical forest strata.

Phlebotomines (Diptera: Psychodidae) are insects worthy of medical importance mainly because some species are the proven vectors of *Leishmania* protozoans (Kinetoplastida: Trypanosomatidae), the etiological agents of leishmaniases (Ready 2013).

The life cycle of *Leishmania* involves close ecological interactions between vector and reservoir systems and niches which are often associated with silvatic ecotopes (Lainson and Shaw 2010). However, progressive changes in transmission patterns may occur either naturally or anthropogenically. In Brazil, several cities have experienced the occurrence and expansion of both visceral leishmaniasis (VL) and cutaneous leishmaniasis (CL) (Miguel et al. 2019). In the Belém Metropolitan Region (BMR), Pará State, CL is occasionally endemic, where the *foci* usually comprise forest fragments inhabited by anthropophilic phlebotomine populations (Ferreira et al. 2014), an essential condition for the establishment of *Leishmania* enzootics (Lainson and Shaw 2010). The etiology is attributed to the following species: *Leishmania* (*L*.) *amazonensis*, *L.* (*Viannia*) *lainsoni*, and *L.* (*V.*) *lindenbergi*, the latter accounting for approximately 40% of cases (Gonçalves et al. 2020).

*Nyssomyia antunesi* is an anthropophilic phlebotomine species widely distributed in South America, mainly in the Amazon Basin, and has been recorded in eight countries, including Brazil, where it occurs in ten Federated States (Aguiar and Vieira 2018). *Ny. antunesi* started gaining medical attention in the Pará State from the 1980s onward, when the first natural *Leishmania*-like suprapylaric infection was registered in this species in a VL endemic area of Marajó Island (Ryan et al. 1984). In the BMR, *Ny. antunesi* was also known to be infected with an unknown trypanosomatid (Silveira et al. 1991). Later, in the same region, it was regarded as a suspected vector of *L.* (*V.*) *lindenbergi* owing to being, by far, the most abundant anthropophilic species in the type locality of this parasite (Silveira et al. 2002). Since then, and chronologically congruent with the popularization of molecular detection techniques in vector ecology, *Leishmania* spp. DNA has been habitually found in *Ny. antunesi* (Vásquez-Trujillo et al. 2008, Trujillo et al. 2013, Thies et al. 2013, Ogawa et al. 2016, Leão et al. 2020, Araújo-Pereira et al. 2020, Da Silva Costa et al. 2021).

The arthropod-borne and zoonotic nature of *Leishmania* life cycles places the investigation of phlebotomines as vectors and mammals as their potential reservoir hosts on the priority list of epidemiological surveillance strategies. Currently, vector and parasite associations are inferred by tracking *Leishmania* DNA in phlebotomines. Among the eligible genomic regions, the mini-exon gene is considered to be unique and tandemly repeated. Moreover, the nontranscribed spacer region is distinct in length and sequence among *Leishmania* species (Fernandes et al. 1994). Likewise, identifying phlebotomine blood meal sources can be an alternative way to unravel potential *Leishmania* reservoirs (Roque and Jansen 2014, Kocher et al. 2017). Mitochondrial DNA genes, such as cytochrome b (cyt b), have been widely used as molecular markers, showing sufficient interspecific variation to distinguish between vertebrate host samples while exhibiting minimal intraspecific variation (Boakye et al. 1999, Steuber et al. 2005).

The present work aimed to provide knowledge of the blood feeding sources of *Ny. antunesi* from an urban park in the Brazilian Amazon region. *Leishmania* DNA detection in the blood was also attempted.

## Materials and Methods

Samples of the present study were derived from an earlier survey conducted in an urban park in the Belém Metropolitan Region, Bosque Rodrigues Alves, Jardim Botânico da Amazônia (BRAJBA), in which the ecology of phlebotomines has been previously studied (Sánchez-Uzcátegui et al. 2020). The BRAJBA has a total area of 15 hectares, being 80% covered by primary forest with about 5,000 trees belonging from 300 species; vertebrate fauna account about 435 specimens, being 29 species living in captivity, and another 29 in freedom/semifreedom conditions (PMB 2015). Entomological captures were performed in the forest environment during four monthly occasions, from January to December 2018, with CDC light traps (*n* = 4) set from 6:00 p.m. to 6:00 a.m. about 1.5 m above ground (*n* = 2) and about 20 m above ground (*n* = 2) in the canopy strata, and aspiration of tree bases from 6:00 a.m. to 8:00 a.m.

Engorged females of *Ny. antunesi* captured were stored for further analysis. They were identified based on the external characteristics and morphology of female spermathecae (Galati 2018), dissected under fresh conditions, and/or slide-mounted in Berlese fluid. The *Ny. antunesi* samples destined for processing and analysis consisted of the entire bodies of female specimens that were not dissected in the field, and gut contents from dissected specimens previously examined for *Leishmania*-like flagellates. The samples were stored in 70% ethanol at −20°C until DNA extraction.

DNA from *Ny antunesi* females was extracted following a protocol adapted from Solano et al. (1997). Samples stored in ethanol were washed briefly in sterile distilled water. Then they were individually macerated within polypropylene tubes and homogenized with 100 µL of 5% Chelex 100 resin beads (Sigma-Aldrich). Each tube was heated at 95°C for 15 min and centrifuged at 13,000 rpm for 10 min. The supernatants were used for PCR.

Quality control of DNA extraction from each entire body sample was performed via amplification of a 540 bp fragment of the dipteran cytochrome b gene from the mitochondrial DNA (mtDNA) using the primers CB3-PDR (5ʹ-CA(T/C)ATTCAACC(A/T)GAATGATA-3ʹ), N1N/PDR (5ʹ-GGTA(C/T)(A/T)TTGCCTCGA(T/A)TTCG(T/A)TATGA-3ʹ), under PCR conditions described by Ready et al. (1997).

*Leishmania* DNA was searched in the samples of gut contents and dipteran cyt b-positive entire bodies, through the amplification of a fragment of the mini-exon gene repeat with the primers S1629 (5ʹ-GGGAATTCAATATAGTACAGAAACTG-3ʹ), S1630 (5ʹ- GGGAAGCTTCTGTACTTTATTGGTA-3ʹ), under PCR conditions described by Fernandes et al. (1994), which could distinguish the *Leishmania* ‘complexes’ based on the differences in their amplicon lengths, as follows: New World dermotropic species subgenus *L.* (*Leishmania*) (330 bp); New World dermotropic species subgenus *L.* (*Viannia*) (250 bp); Old/New World viscerotropic species (450 bp). The positive control consisted of DNA from *L.* (*L.*) *amazonensis* (IFLA/BR/1967/PH8), *L.* (*L*.) *infantum chagasi* (MHOM/BR/1974/PP75(M2682)), and *L.* (*V.*) *braziliensis* (MHOM/BR/1975/M2903).

DNA from gut contents and dipteran cyt b-positive samples were subjected to PCR to amplify a 360 bp fragment from the conserved region of the cyt b gene in vertebrate mitochondrial DNA, using the primers cytb1 (5ʹ-CCA TCCAACATCTCAGCATGATGAAA-3ʹ), cytb2 (5ʹ-GCCCCTCAGAATGATATT TGTCCTCA-3ʹ), under PCR conditions described by Steuber et al. (2005).

The amplified products were visualized by horizontal electrophoresis on a 1% agarose gel with ethidium bromide (0.5 mg/ml) staining. The products were recorded using an L-PIX STi (Loccus) electrophoresis gel photodocumenter.

The vertebrate cyt b PCR products were purified using a commercial kit (Wizard SV Gel and PCR Clean-up System) and quantified using a Quantus Fluorometer (Promega). Samples with more than 10 ng of DNA were sent for sequencing, in duplicate, by the Sanger method, using the ACTGene Análises Moleculares LTDA UFRGS/RS sequencing service. The alignment of the sequences, as well as the electropherograms, was analyzed using BioEdit 7.2.5 software (Hall 1999). The electropherograms were manually checked to remove primer residuals and trim noninformative segments. Consensus sequences were assembled, submitted to GenBank, and assigned the following accession numbers ON316828-ON316840.

Sequences generated were compared with those available in the National Institutes of Health (NIH) genetic sequence database GenBank (Benson et al. 2013), using the nucleotide Basic Local Alignment Search Tool (BLASTn) (Altschul et al. 1990). Sequences with query cover and percent identity values above 97% were considered reliable for association with the top hit ID organism. However, when the occurrence of a given vertebrate was well documented in the study area, identity values above 90% were considered acceptable.

Maximum likelihood (ML) phylogenetic inference was conducted to improve the taxonomic determination of the target sequences. Sequence alignments were performed using the ClustalW algorithm (Thompson et al. 1994) hosted in MEGA X 10.1.6 software (Kumar et al. 2018). To assess the clustering pattern, an ML phylogenetic tree was constructed using the alignments of the generated sequences, together with those of the top hit from the BLAST searches and that from the hot creek toad *Bufo montfontanus* (Anura: Bufonidae) (MK284968.1), to the root as an outgroup. The following configurations were applied: a general time reversible substitution model with gamma distribution rates and the nearest neighbor interchange heuristic inference method, using a consensus bootstrap of 1,000 replications. Clades with bootstraps >90 were considered consistent (Dhar and Minin 2016). Clades were illustrated with VectorStock silhouettes.

## Results

**Fig. 1.**
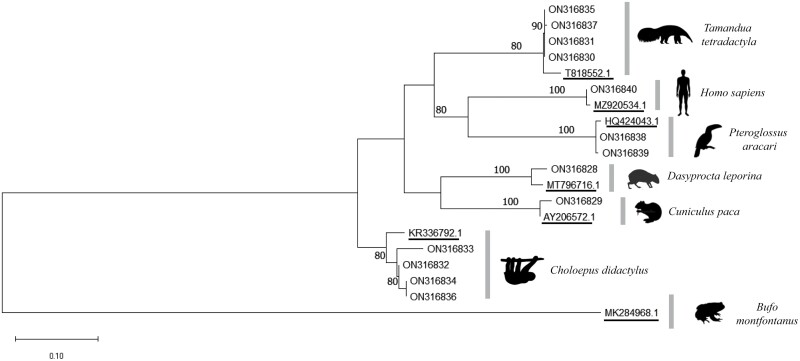
Maximum likelihood phylogenetic tree of partial vertebrate cytochrome b sequences of the phlebotomine *Nyssomyia antunesi* blood feeding sources from the Bosque Rodrigues Alves - Jardim Botânico da Amazônia (BRAJBA), Belém, Brazil, together with their respective identity top-hit from the BLAST searches (GenBank), rooted with *Bufo montfontanus*. Reference accession numbers are underlined. Values at the nodes represent bootstrap support of the ML tree (only nodes with >70% support are presented).

Amplification of dipteran cyt b was positive in 42/59 entire body samples, of which 16 were vertebrate cyt b positive and four samples had sufficient DNA for sequencing. The gut contents of 19/22 females were vertebrate cyt b-positive, and 12 samples had sufficient DNA for sequencing. Therefore, 16 samples were sequenced, 13 of which were informative, and exhibited similarities with the following six vertebrate species: red-rumped agouti, *Dasyprocta leporina* (Rodentia: Dasyproctidae) (1); lowland paca, *Cuniculus paca* (Rodentia: Cuniculiade) (1); anteater, *Tamandua tetradactyla* (Pilosa: Myrmecophagidae) (4); sloth, *Choloepus didactylus* (Pilosa: Megalonychidae) (4); toucan, *Pteroglossus aracari* (2); and man, *Homo sapiens* (Primates: Hominidae) (1). The samples of *D. leporina* and *C. paca* were obtained from the CDC canopy, whereas the others were obtained from aspiration on tree bases (Table 1). Phylogenetic ML reconstruction generated well-supported clades, which were in agreement with the BLAST-based identification of our generated sequences of blood sources of *Ny. antunesi* (Fig. 1). The 42 dipteran cyt b-positive samples and 22 gut contents were submitted for *Leishmania* DNA detection through amplification of the mini-exon gene region. No samples were found positive.

**Table 1.** Vertebrate bloodmeal sources of engorged field-collected *Nyssomyia antunesi* females from the Bosque Rodrigues Alves - Jardim Botânico da Amazônia, Belém, Pará State, Brazil (2018)

*N*	Capture method	Sample	Bloodmeal source	Accession number (generated sequences)	Query coverage (%)	E-value	Identity (%)	Accession number (top hit)
1	CDC canopy	Entire body	*Dasyprocta leporina*	ON316828	98	1e-129	96.7	MT796716.1
2	CDC canopy	Entire body	*Cuniculus paca*	ON316829	99	4e-78	92.42	AY206572.1
3	Tree bases	Gut content	*Tamandua tetradactyla*	ON316830	98	8e-100	99.5	KT818552.1
4	Tree bases	Gut content	*Tamandua tetradactyla*	ON316831	98	8e-100	99.5	KT626616.1
5	Tree bases	Gut content	*Choloepus didactylus*	ON316832	99	3e-94	98	AF232012.1
6	Tree bases	Gut content	*Choloepus didactylus*	ON316833	100	2e-85	96.02	AF232012.1
7	Tree bases	Gut content	*Choloepus didactylus*	ON316834	100	1e-103	95.7	AF232012.1
8	Tree bases	Gut content	*Tamandua tetradactyla*	ON316835	99	8e-100	99.5	KT818552.1
9	Tree bases	Gut content	*Choloepus didactylus*	ON316836	100	1e-103	96.6	AF232012.1
10	Tree bases	Gut content	*Tamandua tetradactyla*	ON316837	98	8e-100	99.5	MW752306.1
11	Tree bases	Gut content	*Pteroglossus aracari*	ON316838	100	4e-98	100	HQ424043.1
12	Tree bases	Entire body	*Pteroglossus aracari*	ON316839	100	4e-98	100	HQ424043.1
13	Tree bases	Entire body	*Homo sapiens*	ON316840	99	2e-141	97.9	LC088152.1

## Discussion

In the present study, we identified blood feeding sources and attempted to detect *Leishmania* DNA in *Ny. antunesi* samples captured in a preserved environment, with diverse and already described fauna and flora, surrounded by urban areas (PMB 2015); this phlebotomine species is recognized as the dominant species in both canopy and ground strata (Sánchez-Uzcátegui et al. 2020). Preserved forest environments, such as the sampled area, generally offer a high variety of blood meal sources for phlebotomines, guaranteeing the maintenance of the gonotrophic cycle (Lainson et al. 1981). Blood feeding sources on a given phlebotomine species collected from numerous widely dispersed areas over a long period must eventually provide some indication of the most likely reservoir(s) of *Leishmania*, especially if they are related to the simultaneous detection of the parasite in dissected phlebotomines from the same area at the same time (Lainson and Shaw 1979).

The identification of bloodmeals from phlebotomines using partial sequences from PCR products of the mtDNA cyt b gene is suitable and widely used for this purpose (Jiménez et al. 2013, Carvalho et al. 2017, Pereira Junior et al. 2019, Doe et al. 2020, Leão et al. 2020, Remadi et al. 2020, Lozano-Sardaneta et al. 2021, Rodrigues et al. 2021, Torchitte et al. 2020). Six vertebrate species were found to be blood feeding sources of *Ny. antunesi*, all of which have already been documented in the BRAJBA (PMB 2015). The eclectic blood feeding repertoire observed for this phlebotomine species is consistent with recent literature. In the rural environment of the western Amazon, it has been found that females from the *Ny. antunesi* obtain bloodmeals from the following vertebrates: *Bos taurus* (Artiodactyla: Bovidae), *Pecari tajacu* (Artiodactyla: Tayassuidae), *Plecturocebus bernhardi* (Primates: Pitheciidae), *Philander canus* (Didelphimorphia: Didelphidae), *Sus. scrofa* (Artiodactyla: Suidae), and *T. tetradactyla* (Da Silva Costa et al. 2021), with the former also being a blood source for *Ny. antunesi* in a closely related region (Pereira Junior et al. 2019).

The most common blood feeding sources were two mammals of the order Pilosa: *T. tetradactyla* and *C. didactylus*. Both have recognized roles as *Leishmania* spp. reservoirs (Roque and Jansen 2014, Muñoz-García et al. 2019), with particular ecological associations with other phlebotomines of the genus *Nyssomyia*. Anteaters spend much of their time climbing up and down trees in search of termite nests and therefore, come into intimate contact with tree trunks inhabiting phlebotomines, such as *Nyssomyia umbratilis* and *Nyssomyia whitmani* (Lainson et al. 1981). In the same way, sloths have been proven as the primary blood feeding sources of *Ny. umbratilis* and *Nyssomyia anduzei* (Christensen et al. 1982).

Two vertebrate cyt b sequences were obtained from toucan. The role of birds in the life cycle of phlebotomines has been discussed, as their presence may influence fly population dynamics with increasing population density, and consequently contribute to *Leishmania* transmission dynamics (Teodoro et al. 1993, De Ávila et al. 2018). In the studied area, *Ny. antunesi* is a canopy-dominant species (Sánchez-Uzcátegui et al. 2020), spatially congruent with birds ecotopes. Similar results were observed with the also canopy dwelling *Ny. umbratilis* in the Guianan Amazon biome, where bird blood were detected as food sources (Vasconcelos dos Santos et al. 2018).

Two vertebrate cyt b sequences were from *C. paca* and *D. leporina*. These ground-dwelling rodents are common blood sources for phlebotomines (Kocher et al. 2017). In addition, *C. paca* is the only known potential reservoir of *L.* (*V.*) *lainsoni* (Roque and Jansen, 2014), a parasite species found in the enzootics of the studied area, but associated with other phlebotomine species, *Trichophoromyia brachipyga* (Sánchez-Uzcátegui et al. 2020, Vasconcelos dos Santos and Silveira 2020).

One vertebrate cyt b sequence belonged to the human genome, strengthening the well-documented anthropophilic behavior of *Ny. antunesi* (Silveira et al. 2002, Pereira Junior et al. 2019, Sánchez-Uzcátegui et al. 2020).

Arias et al. (1982) stated that the vertical habitat of a given phlebotomine species tends to be spatially congruent with that of its associated natural hosts. However, given the small sample size of the present study and considering that some blood sources, such as anteaters, sloths, and birds, frequently move between the canopy and ground level, it was not possible to determine whether feeding occurred arboreally (Leão et al. 2020). Conversely, DNA from terrestrial rodents has been found in *Ny. antunesi* females collected in the canopy, making it impossible to determine whether this phlebotomine species favors bloodmeals from a specific stratum. Based on these findings, it can be assumed that *Ny. antunesi* can feed on the ground and climb into the canopy.

Successful identification of blood feeding sources may have been prejudiced because of the small volume of blood and the time elapsed after feeding. Owing to the quick blood digestion process inherent to dipterans, only recently engorged phlebotomines are suitable for DNA analysis (Sant’Anna et al. 2008). Minuscule amounts of blood are ingested by phlebotomines (Rogers et al. 2002) and, especially in partially fed specimens, there may be insufficient DNA for detection. Moreover, the sizes of phlebotomines vary between species, and those from *Nyssomyia* are usually very small when compared, for instance, with *Lutzomyia* and *Trichophoromyia.* Despite these facts, most of the dipteran cyt b-positive samples were obtained from gut contents, instead of entire bodies, all of which were stored since 2018 to be processed in 2021–2022. The easy handling and long-term stability of DNA in blood samples make this methodology applicable in field conditions, where biological material is often collected far away and processed after a long time (Sant’Anna et al. 2008).

*Leishmania* DNA was not detected in either the entire body samples (dipteran cyt b-positive samples) or the gut contents, which is in accordance with previous reports, where no *Ny. antunesi* from BRAJBA were found to be naturally infected, despite other phlebotomine species known to harbor *Leishmania* at that site, with a general infection rate of 0.013 (Sánchez-Uzcátegui et al. 2020). All gut contents submitted to PCR were previously examined in the field and found negative for promastigotes. However, given the high sensitivity of PCR, the samples were re-examined to detect DNA tracks of dead and disintegrated promastigotes, not viewable through microscopic observation.

True vector importance of *Ny. antunesi* remains uncertain in BMR. However, the present results provide an advance in the biological knowledge of this phlebotomine species, revealing an eclectic and opportunist blood feeding behavior, including attraction to birds and various orders of mammals, these last ones acting as potential reservoirs of *Leishmania* species, distributed throughout the vertical forest strata.
